# On Hypochondriasis
*Confessions of a Hypochondriac; or, The Adventures of a Hyp. in Search of Health." By M. R. C. S. Saunders and Otley. London, 1849. Pp. 310. Small 8vo.


**Published:** 1850-01-01

**Authors:** 


### THE JOURNAL

OF

### PSYCHOLOGICAL MEDICINE

AND

### MENTAL PATHOLOGY.

JANUARY 1, 1850.

### Analytical Reviews.

### Art. I. — ON HYPOCHONDRIASIS.*

Hypochondriasis, the poor man's scourge and the rich man's plague, cowers beneath the cloak and cowl of self-loathing and disgust of life. It is an egotism both mental and bodily, languishing in a sphere of its own creation, apart from the busy bustle of the crowd strolling along the pleasant plains of earth, and averse to everything except the bitter consciousness of its own all-absorbing sense of perpetual solitude and woe!

The melancholy that weighed upon the disgraced premier in Gil Blas and wore away his spirits, presented itself in the shape of a frightful spectre that was never absent from his Grace's disordered vision. It was the forerunner of his end; and though, as Le Sage somewhat maliciously insinuates, his death was inevitable from the moment that the two celebrated physicians from Madrid (who had the merit of curing their patients *quelquefois*), together with their two obsequious executioners, the surgeon and apothecary, set foot upon the threshold; yet the spectral illusion alone was sufficient to signify the nature of his malady, and give warning of his approaching dissolution. We were once acquainted with a Baronet in a distant part of England afflicted in the same mournful way. The village doctor was puzzled, and the ablest heads from the

* "Confessions of a Hypochondriac; or, The Adventures of a Hyp. in Search of Health." By M. R. C. S. Saunders and Otley. London, 1849. Pp. 310. Small 8vo.

metropolis were baffled by these unwelcome visitors, who took departure only upon the decease of their helpless victim. In such cases the nerves are unstrung, and their tone and harmony have been disturbed by a long-continued abuse of the great business or pleasures of the world; for melancholy is one of the penalties paid by "vaulting ambition" for the much envied liberty of being allowed to scale the difficult *itur ad astra*. A friend of ours, of an habitually melancholy mood, was once, in a fit of absence of mind, walking up and down a room, at the bottom of which was a large plain mirror. Suddenly raising his eyes, he beheld his own full-length portrait, and, mistaking it for that of his brother, whom he strongly resembled, reached forward in haste, and shivered the glass in the thoughtless attempt at shaking hands with the image reflected before him. He was roused from his trance by the blow, and taught by a painful reality that there was no one present but himself. Little children are sometimes melancholy, although Heberden affirms the contrary; but they are scrofulous subjects, and so are melancholy young ladies, the Rosa-Matilda's of Lord Byron, himself a prey to the most inveterate sadness. Genius is generally melancholy, but what if there is no genius without scrofula? Johnson, the philosopher of Fleet Street, was atrabilious, Milton amaurotic, Grey gouty, and the interesting Cowper a coward almost to madness. The inimitable Pope, the poet, had a crooked spine, and Torquato Tasso, whose splendid vision of the *Jerusalemme Liberata* ranks among the first of epics, was the sport of a temper tinged with a sadness of the deepest die. It was the same with Dante, and if Shakspeare was not melancholy himself, he at least felt with delicacy the full meaning of the words that he has so appropriately placed in the mouth of Jaques.

But what is genius?—a supernatural gift; sublime because it is perpetual, and enchanting because it embodies in music, verse, bronze or marble, the everlasting beauties of nature. It is ethereal, because it is ideal. The discovery of America, or the conception of a Parthenon, the passage of a Mount St. Bernard, or the *Agnus Dei* of a Mozart, are the creations of a single mind incapable of propagation, and energetic only during the life, or it may be during part of the life of a solitary individual. Homer, who addresses himself to a universe, holds no commerce with any generation of poets; and a Rafaelle or a Carlo Maratti paint in an atmosphere where meaner talents can scarcely draw their breath or handle their brush with freedom and effect. The flame of genius burns alone, the envy or admiration of others, if not an exhausting ecstasy to its hapless possessor. Meteoric in its essence, it shines by fits and starts, appearing on a sudden, flashing for a while, and then expiring.*

Modern sentimentalism, the mimic of genius, is a form of hypochondriacism ; and much of the mawkish literature of the day, surcharged with love-tales, melodramatic escapades, and the wild career of ill-regulated passions, argues a condition of nerves in the writers of such popular nonsense little short of softening of the brain, or at least of some vicious degeneration of the nervous tissues. It leads to the worst of consequences. For, physically speaking, it is a symptom of national or personal decay; and, morally speaking, it inflicts a judicial blindness in the exalted perception of truth, virtue, and every kind of heroic action. To benefit, not only the individual in particular, but society at large, and to afford a substantial means for carrying forward every practical purpose in the regulation, the amelioration, the consolidation, and, indeed, the absolute happiness of an entire community, requires a vigorous and well-governed mind depending upon a temperate, healthy, and strictly disciplined habit of the body.

Heberden, who has drawn a faithful picture of this malady, considers hypochondriasis and hysteria as different forms of the same disease. Few persons are so blessed as to be entirely ignorant of it, or not to have sometimes felt languor and despondency without any manifest cause. It may be of short continuance, and pass away without being noticed by others. But in its severer forms, it is a sort of dream, which, though a person be otherwise in sound health, makes him feel symptoms of every disease ; and, though innocent, yet fills his mind with the blackest horrors of guilt. These lackadaisical people are a sort of timid lambs with the propensities of a bear—*moutons enragés,* according to the facetious expression of a French writer.

The causes of hypochondriasis are as various as they are inexplicable; while sometimes, in direct opposition to the rules of logic, the disease seems to be an effect without a cause. Debility, or a deeply-seated scrofulous taint, appears to reside in most instances at the root of the evil. It precedes and accompanies gout, and is cured by podagra in the great toe. It is observable among the members

* The birth-place of mere scientific genius was Greece. Rome procured from thence her physicians, architects, painters, musicians, &c., whom she paid and despised. The Gauls, Germans, Spaniards, &c., were, like the Greeks, her subjects also, but then those barbarians knew the use of the sword, and the Romans respected them for it. *Græculus* was the stinging word of contempt applied to the Athenians, who, with all their wit, could not manage to retort a similar insult on their masters.

of the same family, who sometimes become maniacal instead of podagrous. It is a symptom of rachitis, and those who suffer from disease of the bones of the spine, are constantly more or less *hyp'd.* A person, whom we attended in his youth for several obscure ailments, was seldom otherwise than melancholy. To mend his fortune he went to Australia, where he succeeded beyond his expectations; but at length died paralysed and comatose. On the examination of the body after death, a spicula of bone was discovered growing from the inside of the calvarium, piercing the dura-mater, and penetrating the brain. Stagnation in the functions of the liver is proverbial for giving rise to the vapours, spleen, melancholy, or black bile. The jaundiced eye is said to look at all things with a sour visage. Excessive fasting as well as eating to excess, like all extremes, produces similar results; and you may starve your victim into sadness on a bare board, as surely as you may over-nourish him with despondency from the enticing viands of too fat a repast. Repletion and hunger are equally the authors of crime, animosity, ill-temper, and an invincible disgust of life.

The mucous membrane of the stomach alone will, when slightly inflamed and acid, prove an obstinate source of hypochondriasis; and, perhaps, sensitive people who can never look another in the face, nor utter a word of their own without being covered with blushes, are the subjects of habitual dyspepsia. A gentleman who lived freely was advised to relinquish his wine and become a tee-totaller: he did so, and went to Paris, where, in a fit of despair, he cut his throat. On a *post-mortem* inspection, an ulcer was found in his stomach. Deranged or abused generative function is another very frequent cause which blanches the cheek, renders the eye wan, exhausts the brain, fixes a sullen sadness on the brow, and tempts almost irresistibly to suicide, or to the perpetration of some of those enormities that every now and then harrow the feelings and shock the good sense of the world. The abuse of mercury for the cure of specific disease is another direful infliction that produces the very worst description of mental suffering, and has ere now instigated many a mutilated reprobate to fill up the measure of perdition by cutting short with his own hand the forlorn remainder of his days. Solitude does the same; and so does an incessant round of dissipation, in company with the giddy throng who waste the fleeting moments of their time in the restless, vain, and insatiable pursuit of sensual enjoyment. Pleasure begets remorse, remorse despair, and despair woe.

Among the many other very learned definitions so scholastically explicated and charitably bestowed upon our fallen race, man is *par*

*excellence* said to be a social or gregarious animal. Few, if any, have ever dared to live alone, any more than they have ever wished to live continually with others without occasional loneliness. Parent-du-Chatelet says, that in the *Maison du Bon Pasteur*, the deaths were 1 in 10, whereas the mortality in Paris was, at the same age, 1 in 75. Persons who, like these unfortunate inmates, had been accustomed to a gay and unrestrained manner of living, sank beneath the comparative solitude and monotonous regularity of a more virtuous and religious *régime*. Habit is everything: and the sudden relinquishment of the worst of vices is likely to bring about the worst of consequences. The *juste-milieu*—the *via media*—the golden mean —where is it?

Actual vice apart, there is nothing in this world worthy of either joy or grief; for, strictly speaking, success and failure are equivalent terms; and the last state of experience is to receive all that happens without emotion, and to regard events with a cool, deliberate, and dispassionate eye. Too serious a reflection on the transitory nature of earthly goods, is more than enough to drive any one crazy, unless he be blessed with a constitution congenitally apathetic, stoical, or extremely religious. But religion itself is, when abused, a powerful source of hypochondriacism. For either it is believed and disobeyed, which gives rise to reproach of conscience; or else it is believed in a wrong sense, whereby the terrors of Divine justice are made to supersede the promises of mercy and forgiveness; although, when received in its true sense, according to the rule of faith, religion is a charm that sweetens everything. *O Potestas, quid non præstas homini?*

The confessions of a hypochondriac, which we have placed at the head of this article, is a humorous description of this unhappy and ignoble class of personages. Fortunately, it has the merit of being written in a pleasing style of narrative which renders the book so enticing that you cannot lay it aside until you have finished it. Light as it may appear at first sight, it is, however, deep enough; for if its surface be sparkling, you may at the same time see to the bottom of it, simply because its depth is clear. The buffoonery is indispensable to its success, since it would be impossible to treat the subject in any other way than that of the burlesque—aiming a random shot at folly as it flies.

The hypochondriac himself, the poor descendant of an affluent family, is restored to wealth by the good luck of a will made in his favour. Without occupation, or rather without the necessary "bore" of earning his daily bread, he quickly becomes tired of himself, of everybody, and of everything. Collecting his bag and baggage, which consists of his own dear self and a very sinister waiting man, or gentleman's gentleman, he resorts to Malvern to try its invigorating air and celebrated waters. Driven from thence by an odd but amusing accident, he undergoes the Hindoo shampooing, and consults a prosperous homœpath at Brighton. The fresh sea-breezes and the fragrant downs are, in his morbid estimation, nothing to be compared with the infinitesimal doses of the one and the scalding manipulations of the other. Hydropathy next has its turn—wet sheets, a crystal beverage, the cool diet, and the presumptive hopes of a water-cure. From these delusive remedies he rushes away in disgust, and recklessly flings himself into the arms of a mesmeri*zer* or mesmeri*zess*—for we believe that as yet the necessary gender is not determined. This, perhaps, is the most *piquante* scene of the whole, and it is told in a very animated and effective manner. Wishing to be put *au fait* of the *science* or *séance*, he abandons himself without reserve to the truth or falsehood of this black or lucid art. Allowing no scruple to hinder him, nor the intrusion of any preconceived idea to bias his sentiments, he frankly delivers himself up, bound hand and foot, to the magic demonstrations of the animal magnetizer; and the penalty he pays for the thorough conviction of his credulity is a public exhibition of himself, if not by name, at least in *propriâ personâ*, within the fashionable saloon of some celebrated person devoted exclusively to mere obscure, recondite, and questionable experiments. Unluckily for the cause of mesmerism, the hypochondriac being convinced of *clairvoyance*, departs full of the gifts of this inward perception which dreams of truisms when fast asleep, but is in fact asleep to the truth when wide awake. "I could not," he says, "repeat the ceremony. The audience were both pleased and astonished; and as they filed off in a crowd, some one, I fancy a duchess, with more money than sense, put a guinea in my hand, and being taken by surprise, she slipped away before I discovered her folly, consequently I was under the painful necessity of putting this gratuity into my pocket. She had, probably, mistaken me for a respectable adventurer—a beggar, exhibiting for the company's entertainment."

It being seldom that time is lost by such patients as these in the pursuit of health, the hypocondriac found out a new magician for the restoration of his jaded nerves. He arrived at an enchanted mansion, where, after being introduced to its mysterious owner, submitted to sundry unintelligible operations, and swallowing a copious draught, perhaps of *hachish,* he seemed to be conducted through apartments of Eastern magnificence, until he reached its inmost recesses. Vatheck and the Halls of Eblis are summoned again from the realms beneath—Narkes and Cafour, with waving torches, and Carathis pronouncing her barbarous incantations. Monsters, with one accord, thrust forth their unsightly snouts, and, finding themselves constrained by the potency of charms, open their hideous mouths, and say—"We are yours—what seek you?" "Fiends," answers the hypochondriac, "I conjure you, by your fiery forms—tell me where is health?" "Out yonder," replies the crew in full chorus: "will this content you?—if so, let us depart." "It will," returns the besotted patient, stretching forward to the corridor through which they were pointing with their sinewy claws,—"it will—begone!—I dismiss you—I cannot tarry, for I am bound in the pursuit of health!" The scene changes, and he awakes and finds himself quietly in bed at his hotel.

What has occupied in our pages a few short paragraphs, is in the original drawn out *in extenso*, and charged with the details of a highly entertaining description. The mask is stripped off the mendacious quack, while even the regularly educated physician is compelled to lay aside his cap and gown, and appear in his real character of adulation, pedantry, or tact. Be this as it may, the poor hypochondriac candidly confesses everything he saw and suffered from a designing valet, no small number of out-of-elbow M.D.'s and a host of unprincipled impostors, quacks, and mountebanks.

The medical man's office is ministerial, not executive. He waits upon death, alleviates pain, and assists in the recovery of health. As soon as he pretends to do more than this, he compromises his character for the sake of a little temporary advantage, or sacrifices his patient's welfare to some views of private interest or ambition of his own. The elements of medicine, when applied in practice, according to the approved rules of experience, are sufficient for all the purposes of life; and if an impatient public be prone to transgress the prescribed limits of reason, it is because they cannot brook the notion of death being the ordinary termination of disease in the same sense as old age is the inevitable result of youth and manhood. Consequently, the professors of the healing art can never hope to escape the shafts of satire, nor have charlatans ever been wanting to lend their countenance to the vulgar prejudices prevailing against them—from the recent days of St. John Long, back to those of Dr. Sangrado, and from the era of the Sangrado of Gil Blas, as far back as the times of the Roman emperor, Lucius Verus, whose dying words were *Turba medicorum Cæsarem perdidit.*

When the nervous system is healthy and highly organized, as it is in some favoured individuals, it is scarcely possible for the disease called hypochondriasis to ruffle its imperturbed placidity. Life is, under such delightful circumstances, an enjoyment of itself. The sensations conveyed from without, impinge upon the sensorium a faithful and correct image of all that passes around it, while the feelings from within are disturbed by the troubles of no deranged viscus, nor jarred by the disorders of functions imperfectly discharged, or by local vitality worn out, effete, and verging to decay. On the contrary, the machinery of life moves on in smooth and equal pulsations, like the flowing melody of some eminent composer touched by the hand of a master accomplished in his art. Such a refined character, indeed, is but little understood by men of the world, still less appreciated by the lovers of what is styled "the great and the extraordinary," and, above all, absolutely disrelished by the vulgar, whose tastes are gratified by nothing except by what is startling, exaggerated, rapid, loud, and imposing. The nerves, in this their highest state, are more remarkable for calmness and composure than either for greatness of manner or productiveness of fancy. In youth and maturity it evinces an almost childlike simplicity of behaviour, terribly liable to fall an easy prey to the wicked and the designing, while in old age its chief attribute is wisdom, in the true sense of the word, whereby the world is weighed in the balance of its nothingness, and the happiness of others is most nobly preferred to its own ease and pleasure. Ethereal and unearthly as such beings are, their occurrence is by no means unfrequent in the circles of domestic life, and the moral grandeur of their comparatively perfect innocence, which is the chief security for their own peace of mind, rises up as an impregnable barrier against the aggressions or desires of depravity, malice, and vice.

History, private and public, sacred and profane, teems with instances of the joyless mood, from the faintest tinge of blue to the darkest stain of inky blackness. It is not easy to account for Domitian's petty passion for spiking flies with a needle, which gave rise to the witticism of Viberias Crispus, who, being asked if any one was with the Emperor, replied, "No, not even a fly!"—any more than we can account for the abominations of Heliogabalus, when a buffoon was præfect of the prætorium and signal vice was the grand recommendation to honours and dignities. Perhaps, they are nothing more than some of the trifling particulars set down in the reckoning of human folly, the items of which are interminable. That Egyptian king who built a pyramid procured by his daughter's infamy, could scarcely have been otherwise than broken-hearted; neither could he who sacrificed two children, as Herodotus tells us, to appease the winds, have had a much easier conscience; for human nature is always the same, and iniquity is sure to leave its sting behind. Queen Elizabeth, it is recorded, never smiled after the execution of Essex; and the Protector Cromwell, after having beheaded Charles I., wore a breastplate beneath his hauberk, in consequence, as every one knows, of a pamphlet published at that time, entitled "Killing no Murder." Cleomenes, that *mauvais sujet* of a Spartan, played so many odd tricks in so very excited a manner, that his best friends agreed to place him in the stocks; where, however, in defiance of his discreditable position, he contrived to possess himself of a sword from the hand of a helot, with which he hacked his flesh in pieces till he died. His fate was attributed to various crimes that he had committed, but chiefly to that of sacrilege. The rage of Crœsus upon the death of his son Atys from the wound of a spear prefigured to him in a dream, implies a loss of self-control tantamount to a total loss of reason; and so, likewise, the remorse of Œdipus in tearing out his own eyes, and the infuriated agony of Orestes for matricide, affecting as they may be when exhibited in a classic or tragic point of view, are only excusable on the ground of pagan darkness and unqualified infidelity. But even in the twilight of the world, such punishments were imputed to their proper origin, for the ancients beheld in the fate of these great actors nothing more than the reward that was their due. Hence the poetic justice, so much extolled by the learned, is only true in fiction because it is first of all true in the critical affairs of life. The Greek Emperor, Constans, who from motives of political jealousy put his brother to death, was incessantly pursued, night and day, on land and at sea, by a phantom sprung from remorse of conscience, presenting to his lips a cup of blood, saying, "Drink, brother—drink!"

The spiritual dryness so often mentioned by ascetic writers, and experienced by religious enthusiasts themselves, is apparently a very severe form of hypochondriasis. St. Theresa relates of herself in the third person, that for forty years she had not passed a day without anguish and various kinds of sufferings. St. Catharine of Sienna confessed that she laboured under so heavy a darkness of spirit, that no one could possibly imagine it—she saw herself a hundred times on the brink of a beetling precipice which she was prevented from toppling over by the agency of an unseen hand. In the life of St. Benedict, there is an anecdote told of a certain monk who was tempted to renounce his profession by the spectre of a little black urchin continually beckoning him away from his duties. St.

Francis of Assise was always displeased at the sight of religious sadness, as being the sign of a will much indisposed and a body ill at ease. Drive away sadness, says the wise man, for it hath killed many and there is no use in it.

Antonio Estanez, a physician at Pelonna in Spain, relates that in 1727, a furious delirium prevailed in that neighbourhood, of an epidemic character; and Dr. Weithrecht, physician to the hospital at Vesovia, makes mention of an epidemic madness that broke out in 1767, and attacked no one but foreigners, or strangers to the locality. Epilepsy appeared as an epidemic in Carinthia, in 1717, in the interval between a lunar and a solar eclipse; many sank under it, and those who recovered owed their cure to antispasmodics, or a miliary eruption. The lycanthropy of Avicenna, described by Ovid in the Metamorphosis of Lycaon, king of Arcadia, reigned epidemically at Alkmäest, in 1572. These maniacs ran about barking furiously and aiming blows at every dog they met; and Silimachus (A.D. 100), a follower of Hippocrates, recounts an epidemic nightmare that attacked everybody, and seemed to be contagious. If not treated antiphlogistically, it degenerated into epilepsy. The madness of Nebuchadnezzar is supposed to have been a chronic lycanthropy. Hippocrates, in the fifth book of his Epidemics, mentions a curious nervous affection in a person named Nicanor, who was so terrified at the sound of a flute, that he lost the power of deglutition, particularly at festivals in the night, or evening parties; and he also gives another instance of a similar sort in one Democles, who dared not venture near the edge of a precipice, nor pass over a bridge, nor indeed walk by a dike, lest he should come to some harm. We knew a gentleman who was afraid of passing beneath the shadow of a certain steeple in London, lest it should fall upon him. Lenhopek, according to Feuchsterleben, mentions, a woman whose entire body turned black upon being accused, by her daughter, of a heinous crime; and a philosopher is said to have gone grey in a fit of grief for the loss of a manuscript in a storm at sea. Terror makes the hair stand on end, but sorrow makes the hair straight. Its turning suddenly grey indicates extreme prostration of the vital powers, and the depressing emotions that produce it have been chronicled of old for inexorably bringing down the grey head with sorrow to the grave. Some melancholics have passed their lives in collecting toys, whilst others have squandered their fortunes in designing and constructing figures of a monstrous and ridiculous shape.

Do you see yonder luckless wight, motionless in his chair, with his eyes fixed on the fire, where he is building ideal castles among the smouldering embers before him? He is conversing with himself; and were Dr. Wigan here, he would declare that it was a decided case of duality of mind: one side of his head is larger than the other, and, consequently, one cerebrum is smaller, less energetic, and the less conceptive of the two. Who is he? Nobody; or, at least, in the language of the great world, he is a cipher without a name. And yet the thread of destiny is interwoven with as many various colours in the low as in those of high estate; for each one's life is a novel of its own. Dr. Wigan may be right, for upon the hypothesis of the brain being a two-fold organ, his history is two-fold throughout its course. One brain has always been weaker than its fellow, and the better of the two has had the greatest difficulty in overruling the errors of the worse. When quite a boy he meditated suicide, and selected a deep pond with steep banks for the purpose of drowning himself. He fixed upon the day, and walked to the spot with the deliberate intent of so doing. But when he arrived there, the sun was shining, and the deceitful water looked so clear that he could see the ooze and sludge at the bottom of it. He thought he could have jumped in and have done the deed had the water only been turbid and the day dull. As it was, his stronger brain, or perhaps his good angel, held him back and bade him return. Twice, in after years, he seriously meditated a murder which would have rivalled in horror the atrocities of much more recent date. But again his good angel, or his better brain, interposed and stayed his murderous aim. For years, suicide was never absent from his thoughts, and often did he soliloquize upon the edge of his razor, as he felt its keenness and examined its fitness for the dreadful purpose. Such was the fiery trial of his inward life; but his outward was just the reverse,—steady, regular, frugal, and industrious; till at length his guardian angel, old age, or his better brain, obtained the mastery over his evil propensities, and released him from his lot. See! there he sits, gazing on the fire, wherein he descries, among the ignited coals, a yawning chasm, within which is a glowing cavern leading to a brilliant vista full of diamonds. The crumbling cinders have fallen in and crushed the image,—he starts from his reverie. He rises and looks out upon the scene before him. The brief autumnal day is setting on the dim moors,—there are storms of wind and rain,—the swallows are leaving, or have left,—and the year is on the wane. In the distance lies the gloomy and troubled outline of the sea. "Ah!" he murmurs to himself, "that world of waters! a few years more, and I shall be like the undulations that rose and sunk this morning on the bosom of the deep, and are now lost and forgotten among the countless billows of the ocean! *Pensa, che questo di mai non raggiorna.*

> "Life is not to be dated by years,
> There are moments that act like a plough,
> And there's never a furrow appears
> But has torn up the soul and the brow!"

Saul, the king of Israel, and Alexander the Great, were men of melancholy temperaments, and the wry-neck of the one as well as the extraordinary height of the other, indicates a scrofulous diathesis in both. The moody jealousy of Saul when he sought to transfix David to the wall with a spear was soothed by the means of music, and the dire remorse of Alexander for having slain his friend Clytus with a javelin at a feast was the deleterious effect of wine. They were both of them men of incorrect lives,—the one was a drunkard, and the other a wilful transgressor of the Divine command. Each was a great man in his way; although the dignified reserve, humble birth, regal address, and national policy of the son of Cis surpassed by far the haughty demeanour of the son of Philip of Macedon, as, with admirable military skill, he penetrated the ghauts or passes of modern Affghan, and descended into the plains of the Punjaub, or rode in splendid panoply along the ranks mounted on his fierce Bucephalus. Saul was a hypochondriac to the last, for in a desperate fit he went and consulted a proscribed witch at midnight. To-morrow, said the disturbed shade of the prophet, thou and thy sons shall be with me, at this time; and the next day, as the sun went down, the enemy pressing in hot pursuit on the king's discomfited troops, Saul, together with his armour-bearer and his three sons, was left among the dead on the field of battle. Racked with the paroxysms of an ague, too mighty for cure, Alexander, as he issued his last commands from the banks of the Euphrates at Babylon, counted out the few scanty weeks, days, and hours that still remained to him of all his earthly glory. Melancholy had marked them for its own; and the stories of their lives are left like lofty columns standing alone in the midst of the desolation of ages, in order to admonish a reluctant world that mental disease of the worst description is the invariable result of intemperance, wilfulness, and the indulgence of excessive desires. One is almost tempted to conclude with the idiot, that the world is a large madhouse, and a private madhouse is the world in miniature.

The more deeply we dive into the mysteries of the mind or spiritual being, the more completely do we find ourselves involved in the darkness of a shadowy atmosphere through which are flitting past us phantasms that have no existence in the external world of daylight beyond. Within the wide aerial halls of the soul all is ghostly. Solid things of sight are gone, and nought remains save what material-minded men of earth stigmatize and reject with scorn as visionary, ideal, or unreal; until, as it occasionally happens, they are taken by surprise on the sudden breaking down of the fragile wall of flesh, which unexpectedly exposes to their sight, and perhaps lets loose upon their astonished senses, some of those unreal visionary elements of thought in the substantive shape of hypochondriacism, hysteria, ecstasy, cataleptic somnambulism, or trance; and then they close their eyes against the unwelcome vision, and cry out madness! Little do they imagine that they themselves are such as they behold. A very slight alteration in the equipoise of the nicely-balanced moral and intellectual faculties would easily disarrange their own understandings and transmute them, beyond the power of their will into monsters of crime or folly*—such as, in fact, they are apt to hear of or regard with pity in the well-regulated cells of Bethlem, or within the careful precincts of a more secluded asylum. Many a culprit at the bar of justice is the victim of disease much more than the condign felon of an impartial verdict; and a medical philosopher with the lantern of a modern Diogenes might, among the convicts at Portsmouth, or in any one of our penal settlements, read a tale in the history of each of those unhappy outcasts, so pitiful and pathetic, that, according to the apt hyperbole of the dramatist, "our tears would drown the wind" at the recital of it. We are the prey of circumstances—our usefulness and happiness, nay—our very characters and influence depend upon events over which we have not the slightest control. Our birth, name, lineage, country, epoch, fortune, age, and place, we must receive such as they are bestowed upon us—we must take them, such as they are, for we cannot choose. A misshapen skull, a hump back, a clubbed foot, a defective liver, a weak stomach, and a degenerate set of nerves belong to our family ancestry and descend to us either as a collateral bequest, or as our patrimony by right; and if they prove themselves to be heir-looms or legacies which fail to help us forward in the path of life, we must submit to

* "L'homme a beau vanter dans son orgueil, la supériorité de son intelligence, sur celle des animaux: les maladies mentales qui viennent l'affliger mettent souvent, non pas même au niveau, mais au-dessous de la brute; puisqu'alors sa vie et ses actions sont hors de sa sphere d'existence, au lieu que celle des animaux sont conséquentes à leur manière d'être et ne s'écartent point de l'état dans lequel ils furent créés."—*Histoire des Epidemiques, par Ozanam. 2nde edit. Paris.* 1835, *Tome* iv. p. 255.

the failure and abide the consequences of our innate errors and defects. Society must protect itself: and the *forum judicii* cannot pretend to draw the precise line of demarcation between *actual* transgressions and *possible* imbecility which belong alone to the casuistry or moral theology of the *forum conscientiæ*. Time, the father of experience, has no leisure for deciding subtleties so delicate as these. He divides and swallows down the good and the bad, and—*the world goes on!*

---

